# Pairwise common variant meta-analyses of schizophrenia with other psychiatric disorders reveals shared and distinct gene and gene-set associations

**DOI:** 10.1038/s41398-020-0817-7

**Published:** 2020-05-12

**Authors:** William R. Reay, Murray J. Cairns

**Affiliations:** 1 School of Biomedical Sciences and Pharmacy, The University of Newcastle, Callaghan, NSW Australia; 2 Centre for Brain and Mental Health Research, Hunter Medical Research Institute, Newcastle, NSW Australia

**Keywords:** Genetics, Psychiatric disorders

## Abstract

The complex aetiology of schizophrenia is postulated to share components with other psychiatric disorders. We investigated pleiotropy amongst the common variant genomics of schizophrenia and seven other psychiatric disorders using a multimarker association test. Transcriptomic imputation was then leveraged to investigate the functional significance of variation mapped to these genes, prioritising several interesting functional candidates. Gene-based analysis of common variation revealed 67 schizophrenia-associated genes shared with other psychiatric phenotypes, including bipolar disorder, major depressive disorder, ADHD and autism-spectrum disorder. In addition, we uncovered 78 genes significantly enriched with common variant associations for schizophrenia that were not linked to any of these seven disorders (*P* > 0.05). Multivariable gene-set association suggested that common variation enrichment within biologically constrained genes observed for schizophrenia also occurs across several psychiatric phenotypes. Pairwise meta-analysis of schizophrenia and each psychiatric phenotype was implemented and identified 330 significantly associated genes (*P*_Meta_ < 2.7 × 10^−6^) that were only nominally associated with each disorder individually (*P* < 0.05). These analyses consolidate the overlap between the genomic architecture of schizophrenia and other psychiatric disorders, uncovering several candidate pleiotropic genes which warrant further investigation.

## Introduction

Schizophrenia is a psychiatric disorder which is proposed to arise from a complex interplay between heritable and environmental factors. As a polygenic disorder, the genomic architecture of schizophrenia encompasses variation throughout the genome which have population frequencies ranging from common to ultra-rare^1–4^. Genome-wide association studies (GWAS) of schizophrenia have been able to recapitulate a notable proportion of the heritability estimated from twin-studies, with SNP based heritability in the most recent GWAS calculated to be ~23% on the liability scale, assuming a population prevalence of 0.7%^1^. This polygenic signal is distributed amongst many genes and biological systems genome-wide, and thus, further research is required to fully appreciate the underlying biology captured by GWAS and its relevance to the pathophysiology of schizophrenia.

The diagnostic boundaries between schizophrenia and other psychiatric disorders remain difficult to define. Psychiatric comorbidities are common in patients with schizophrenia^5^, while the defined clinical presentation of the disorder itself resembles that of other diagnoses. For instance, major depressive disorder (MDD) diagnosis is prevalent amongst individuals with schizophrenia^6,7^. However, negative symptoms inherent to schizophrenia, which include avolition and asociality, are also closely linked to MDD despite their classification as distinct clinical entities^7–9^. Collectively, this supports the existence of shared aetiological factors between schizophrenia and the spectrum of psychiatric illness.

**Fig. 1:**
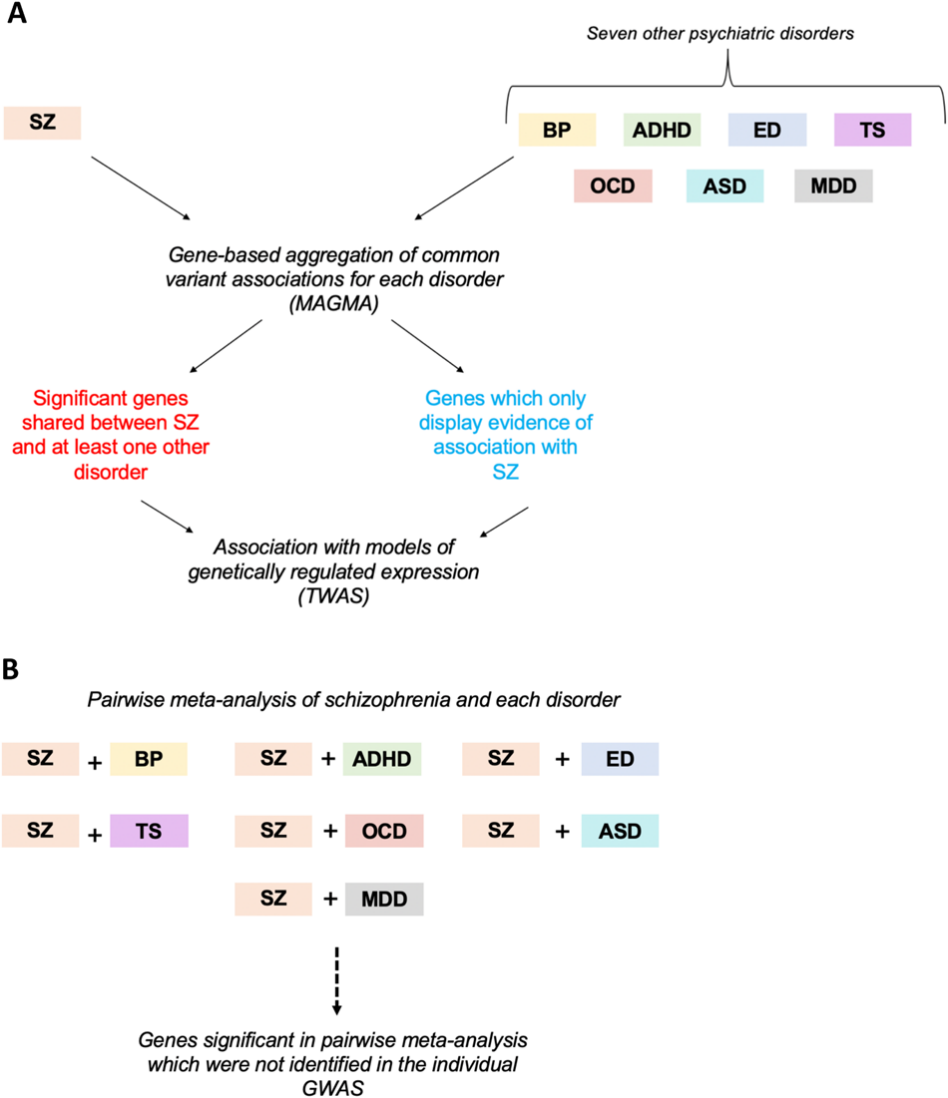
Analysis workflow to investigate shared gene-based common variant associations between schizophrenia and other psychiatric phenotypes. **a** Gene-based aggregation of variant-wise common variant associations was performed using the MAGMA approach for schizophrenia (SZ), along with seven additional psychiatric disorders: bipolar disorder (BIP), attention-deficit/hyperactivity disorder (ADHD), eating disorder (ED), Tourette’s syndrome (TS), obsessive compulsive disorder (OCD), autism-spectrum disorder (ASD) and major depressive disorder (MDD). Genes were firstly selected which survived Bonferroni correction in the schizophrenia dataset along with at least one of the other disorders. Secondly, we identified a subset of genes associated with schizophrenia which did not display uncorrected nominal significance (*P* > 0.05) with any of the other seven phenotypes. Transcriptomic imputation (TWAS) was leveraged to investigate the correlation between imputed models of genetically regulated expression to explore the functional significance of variation affecting the (i) shared genes and (ii) genes only associated with schizophrenia. **b** A pairwise gene-based meta-analysis was performed between SZ and each disorder separately to identify significant genes which were not uncovered by analysing the respective phenotypes individually.

Genomic evidence reinforces this trans-diagnostic paradigm, facilitated by the GWAS now available for a number of psychiatric disorders in addition to schizophrenia^10–16^. Linkage disequilibrium (LD) score regression has demonstrated that schizophrenia displays positive genomic correlation with several psychiatric phenotypes^17^, with the most significant relationship observed with bipolar disorder (BIP, *r*_g_ = 0.7, s.e.m. = 0.020)^10^. However, the biological mechanisms encapsulated by these cross-disorder correlations remain largely uncharacterised. Large-scale post-mortem brain transcriptomic data assembled by the PsychENCODE consortium has characterised differentially expressed transcripts shared between schizophrenia, bipolar and autism-spectrum disorder, although GWAS allows the interrogation of shared genomic risk in much larger samples^18^. Genes and systems associated with schizophrenia which exhibit pleiotropy within psychiatry, that is, a significant relationship with another disorder, may be particularly biologically salient. Further, elucidation of genetic factors specific to schizophrenia may aid in identifying the underlying origin of clinical features which are more distinct to the disorder. Previous cross-disorder association analyses have largely focused on individual SNPs and consider a set of psychiatric phenotypes simultaneously^19,20^. In this study, we implemented a multimarker test of association from a schizophrenia focus in relation to seven other psychiatric disorders—bipolar disorder (BIP), attention-deficit/hyperactivity disorder (ADHD), autism-spectrum disorder (ASD), major depressive disorder (MDD), obsessive compulsive disorder (OCD), Tourette’s syndrome (TS) and eating disorder (ED). We sought to identify pleiotropic genes and gene-sets which were associated with schizophrenia and at least one other of these phenotypes. The workflow for this approach is described in Fig. 1. We uncovered several interesting schizophrenia-associated genes and gene-sets which are shared, along with a subset of genes specifically linked to schizophrenia. Furthermore, we performed pairwise meta-analysis of schizophrenia with each of the phenotypes to identify novel associations.

## Materials and methods

### GWAS summary statistics

The largest European ancestry GWAS with full genome-wide summary statistics available was obtained for schizophrenia (*N* = 105318, Pardiñas et al.^1^) and seven other psychiatric disorders. The other disorders and their respective GWAS were as follows: bipolar disorder (BIP, *N* = 51710)^10^, attention-deficit/hyperactivity disorder (ADHD, *N* = 53293 [European subset])^11^, major depressive disorder (MDD, *N* = 173005) [excluding the 23andMe cohorts for which full summary statistics are not publicly available])^12^, obsessive compulsive disorder (OCD, *N* = 9725)^13^, eating disorder (ED, *N* = 14477)^14^, autism-spectrum disorder (ASD, *N* = 46351)^15^ and Tourette’s syndrome (TS, *N* = 14307)^16^. Schizophrenia summary statistics were downloaded from the Walters group data repository (https://walters.psycm.cf.ac.uk/), while the remaining summary statistics were obtained from the website of the psychiatric genomics consortium (PGC, https://www.med.unc.edu/pgc/results-and-downloads/). A range of phenotypic definitions such as DSM, ICD-10 and electronic medical records were used in the respective GWAS analyses, which further information provided by each of the referenced publications.

### Gene-based association analysis

Gene-based association was undertaken for each of the disorders using the MAGMA package v1.06b (mac) as described elsewhere^21^. Briefly, the gene-based method implemented by MAGMA utilises *P*-values as input, whereby the test-statistic is a linear combination of genic *P*-values. We used the default gene-based test in MAGMA which was the mean of the *χ*^2^ for the variants annotated to each gene. In order to account for dependent *P*-values due to linkage disequilibrium (LD) between variants, the 1000 genomes phase 3 European reference panel is used to derive variant-wise LD such that the null distribution can be approximated. Variants were mapped to 18297 autosomal protein-coding genes in hg19 genome-assembly (NCBI), obtained from the MAGMA website (https://ctg.cncr.nl/software/magma). We removed genes which arise from the extended major histocompatibility complex (MHC, chr6:28477797–33448354) on chromosome 6 due to the complexity of LD in that region, as is standard practice. Genic coordinates were extended 5 kilobases (kb) upstream and 1.5 kb downstream during annotation to capture potential regulatory variation. Statistical inference for a significantly associated gene for each disorder was set as *P* < 2.7 × 10^−6^ to adjust for the number of genes tested via the Bonferroni method.

### Gene-set association analysis

Competitive gene-set association for each disorder was undertaken with MAGMA. This model tests the underlying null hypothesis that genes within a set are no more strongly associated with the phenotype than all other genes^21,22^. MAGMA constructs a linear regression model wherein genic association (transformed to *Z* via the probit function) is the outcome, with adjustment for confounders including gene-size and genic-minor allele count. A one-sided test is performed for the term in the model which specifies whether each gene is within the set of interest (*β*_GS_), such that the null hypothesis is *β*_GS_ = 0 and the alternative *β*_GS_ > 0. We selected 7296 hallmark, canonical and gene ontology gene-sets from the molecular signatures database (MSigDB) for gene-set association^23^. We also tested two hypothesis driven gene-sets which survived multiple-testing correction in the Pardiñas et al. schizophrenia GWAS analyses^1^. Probability of loss of function intolerance (pLI), as a metric of biological constraint, was obtained from ExAC—with all genes displaying high intolerance (pLI ≥ 0.9, *N*_Genes_ = 3230) included in the first set^24^. As constrained genes are postulated to have increased expression in the brain^24^, we also repeated the analysis covaried for the expression of each gene (median transcript per million) in the brain. Genes were annotated using brain RNA-sequencing data (cortex) from the genotype-tissue expression consortium (GTEx v7)^25^. The second hypothesis driven set was obtained from Darnell et al., selecting Fragile X Mental Retardation Protein (FMRP) targets using a stringent false discovery rate (FDR) < 0.01 threshold^26^.

### Transcriptome-wide association studies (TWAS)

TWAS was performed using FUSION (https://github.com/gusevlab/fusion_twas/) with default settings as described previously^27,28^. Summary statistics were formatted using the LDSC framework as is usual practice (munge_sumstats.py, https://github.com/bulik/ldsc), which included the removal of variants with an imputation INFO score <0.9 and variants which were not SNPs amongst other parameters using the default settings^29^. TWAS uses transcriptomic imputation, which builds models of eQTL SNPs to predict (impute) the genetically regulated component of expression. SNP weights from these models were obtained from the FUSION website for models imputed using data from the dorsolateral prefrontal cortex (DLPFC, CommonMind Consortium) and whole blood (Young Finns Study)^30,31^. Statistical inference, to account for the number of models tested, was set at *P* < 9.22 × 10^−6^ (5420 models tested) and *P* < 1.06 × 10^−5^ (4701 models tested) for the DLPFC and blood, respectively. The underlying prinicipal of TWAS is that the expression of genes identified through this method are genetically correlated with the phenotype of interest. Conditional analysis was then undertaken to identify disorder associated genes which are independent versus those co-expressed with a genetic predictor which is shared between them. This was implemented with the FUSION package R script FUSION.post.process with the locus window set at 100,000 base pairs.

### Pairwise cross-disorder meta-analysis

Genic *Z*-scores for schizophrenia were meta-analysed in a pairwise manner with the other seven disorders using the --meta flag in MAGMA. This utilises Stouffer’s weighted *Z* method, in which the *i*th *Z* score is weighted (*w*_*i*_) by the sample size of the respective GWAS:

$$Z_{{\mathrm{meta}}}\sim \frac{{\mathop {\sum }\nolimits_{i = 1}^k w_iZ_i}}{{\sqrt {\mathop {\sum }\nolimits_{i = 1}^k w_i^2} }}$$

Analogous to the univariable gene-based method, the test-statistic follows a standard normal distribution under the null hypothesis^32^. Gene-set association was then undertaken using the model described above with *Z*_meta_ the response variable in each instance. There was limited overlap of controls between the schizophrenia dataset and some of the other phenotypes, however, given the different diagnostic endpoint and summation of SNP-level effects to a gene-based *P*-value, we maintain that this analysis is appropriate for gene discovery.

## Results

### Gene-based association revealed genes specific to schizophrenia and shared with other psychiatric disorders

We implemented a multimarker method to find genes enriched with common variant associations for each disorder. Considering genes outside the extended major histocompatibility complex (MHC) region, 456 genes were associated with schizophrenia below the Bonferroni threshold (*P* < 2.7 × 10^−6^, Supplementary Table 1). The seven other psychiatric disorders also all had at least one significant genic association (Supplementary Table 2), ranging from 121 Bonferroni significant associations for BIP, to just one for OCD (Kit Proto-Oncogene, Receptor Tyrosine Kinase [*KIT*], *P* = 2.3 × 10^−7^).

**Fig. 2:**
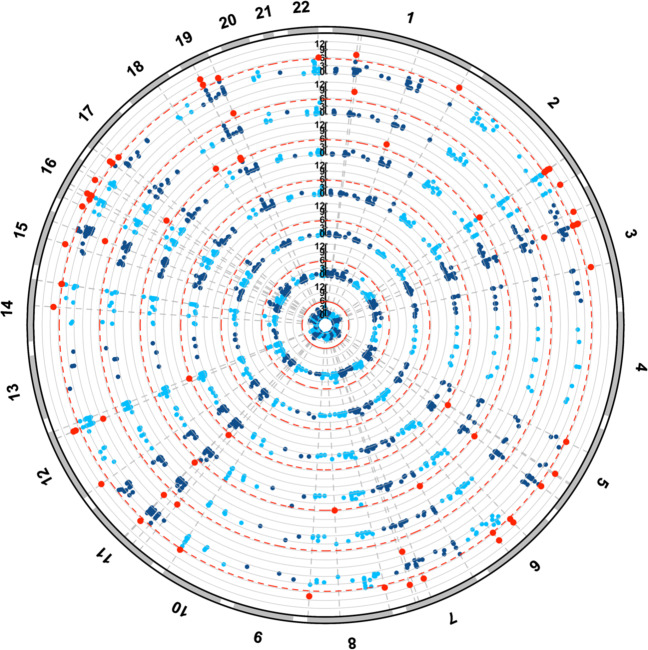
Schizophrenia-associated genes shared with other psychiatric disorders. Association of Bonferroni significant schizophrenia genes with seven other psychiatric disorders. Results presented as a circular Manhattan plot for the −log_10_ *P*-value of association for each gene per disorder. Significant schizophrenia genes by MAGMA which survive correction in each disorder are highlighted red (*P* < 2.7 × 10^−6^). Each shell of the plot represents a different disorder, radiating outwards in the following order: OCD, TS, ED, ASD, MDD, ADHD and BIP.

We investigated the association of the 456 genes which survived Bonferroni correction in the schizophrenia GWAS in relation to the other seven disorders to identify pleiotropic genes (Fig. 2, Supplementary Fig. 2). In total, there were 67 genes significant after Bonferroni correction in the schizophrenia GWAS and at least one additional psychiatric GWAS (Table 1, Supplementary Table 3). *CACNA1C* was the most significant pleiotropic gene in terms of association with schizophrenia, as it also survived correction for the BIP GWAS, *P*_BIP_ = 1.72 × 10^−9^. BIP shared the greatest number of significant genes with schizophrenia—*N*_Shared_ = 47, 39% of all significant BIP genes. Significant genes after multiple-testing correction were also shared between schizophrenia with ADHD (*N*_Shared_ = 8, 32% of all significant ADHD genes), MDD (*N*_Shared_ = 10, 53% of all significant MDD genes), and ASD (*N*_Shared_ = 3, 18% of all significant ASD genes). One gene, Sortilin related VPS10 domain containing receptor 3 (*SORCS3*), was associated with schizophrenia and two of the additional disorders—ADHD and MDD (*P*_SZ_ = 2.91 × 10^−8^, *P*_ADHD_ = 1.51 × 10^−9^, and *P*_MDD_ = 5.66 × 10^−8^).

**Table 1:** The top ten most significantly associated schizophrenia genes by MAGMA which were also significant after multiple-testing correction in at least one other psychiatric disorder tested.

Gene symbol	Shared disorder	*P*_Schizophrenia_	*P*_Shared disorder_
*CACNA1C*	BIP	1.76 × 10^−20^	1.72 × 10^−9^
*TCF4*	MDD	5.68 × 10^−18^	2.24 × 10^−8^
*C10orf32*	BIP	1.30 × 10^−14^	8.26 × 10^−7^
*ZFYVE21*	BIP	3.69 × 10^−14^	1.59 × 10^−6^
*SNAP91*	BIP	3.06 × 10^−12^	1.73 × 10^−6^
*BCL11B*	BIP	6.27 × 10^−12^	5.73 × 10^−9^
*GATAD2A*	BIP	2.30 × 10^−11^	8.85 × 10^−9^
*FGFR1*	BIP	3.37 × 10^−11^	5.03 × 10^−7^
*SFMBT1*	BIP	5.57 × 10^−11^	1.93 × 10^−7^
*MSRA*	ASD	8.95 × 10^−11^	6.94 × 10^−7^

The *P*-value for schizophrenia and the shared disorder are listed.

A transcriptome-wide association study (TWAS) was performed for each of the disorders which displayed at least one shared genic association with schizophrenia (Supplementary Tables 4, 5). TWAS leverages imputed models of genetically regulated expression to identify genes for which expression is genetically correlated with the trait of interest^27^. We aimed to identify genes whose predicted expression is associated with schizophrenia and at least one other psychiatric phenotype. Firstly, we considered models for significant *cis*-heritable genes in the DLPFC; 24 of the 67 candidate pleiotropic genes using MAGMA shared between schizophrenia and at least one other disorder had available models in this tissue. The imputed expression of three genes was associated with both schizophrenia and BIP (*P* < 9.2 × 10^−6^, corrected for the 5420 models tested). In two of these instances, overexpression was associated with both schizophrenia and BIP: *HAPLN4*—(schizophrenia: *Z*_TWAS_ = 5.03, *P* = 4.66 × 10^−7^), (BIP: *Z*_TWAS_ = 5.6506, *P* = 1.6 × 10^−8^)], and *NEK4*—(schizophrenia: *Z*_TWAS_ = 5.34, *P* = 9.25 × 10^−8^), (BIP: *Z*_TWAS_ = 5.13, *P* = 2.9 × 10^−7^). An additional gene, *GLT8D1*, proximally located to *NEK4*, also survived correction the schizophrenia and BIP analyses. Conditional analysis was performed to reveal whether these were conditionally independent. *NEK4* was conditionally significant in both disorders below the Bonferroni threshold, while in this construct *GLT8D1* was only marginally significant for schizophrenia (*P*_Conditional_ = 0.034) and not significant for BIP (*P*_Conditional_ = 0.69). In addition, conditional analyses of the *HAPLN4* locus for schizophrenia suggested that this association was primarily explained by another gene, *GATAD2A*. Interestingly, *GATAD2A* did not survive correction in the BIP TWAS (*P* = 3.8 × 10^−3^), and implicated *HAPLN4*, in contrast to schizophrenia. There are two plausible explanations for this phenomenon: firstly, technical variability may affect the number of loci tagged in this region between the respective GWAS, or secondly, different biological effects may be conferred by variation mapped to this locus in BIP relative to schizophrenia. Further biological investigation is required to elucidate the mechanisms underlying this signal. An additional four shared genes derived by MAGMA were significant after correction in relation to schizophrenia but trended towards significance for BIP (*P*_BIP_ < 9.25 × 10^−4^: *DDHD2*, *TMEM110*, *ITIH4*, *COG8*), while downregulation of *SFMBT1* expression trended towards significance for association with risk for both disorders. Moreover, decreased expression of Mediator complex subunit 8 (*MED8*) was associated with both schizophrenia and ADHD.

Imputed expression models derived from blood data were applied as above, with 22 significant models available. Upregulation of Neuromedin B (*NMB*), for which expression was not significantly *cis*-heritable in the brain, was associated with schizophrenia and BIP risk. *GLN3* (G protein nuclear 3) was similarly significant for schizophrenia and BIP, however, due to its proximity to *NEK4* this may arise from the same underlying genomic signal. In blood, *NEK4* does not display significant *cis*-heritability, required for imputation, and thus, conditional analyses could not be directly performed. A pleiotropic effect for overexpression of Zinc Finger DHHC-Type Containing 5 (*ZDHHC5*) on schizophrenia and MDD was also observed using blood SNP weights—schizophrenia: *Z*_TWAS_ = 5.96, *P* = 2.49 × 10^−9^, MDD: *Z*_TWAS_ = 4.54, *P* = 5.62 × 10^−6^.

### Common variant derived genic association specific to schizophrenia in psychiatry

We then sought to identify genes which were only associated with schizophrenia (Supplementary Table 6). Firstly, the majority of genes associated with schizophrenia (*N* = 390) did not survive Bonferroni correction for any of the seven other disorders. However, as many of these genes trended towards corrected significance in one or more of the phenotypes, we investigated the subset of genes which were not nominally uncorrected significant for any other psychiatric GWAS examined in this study (*P* > 0.05) and identified 78 such genes. The gene encoding Serologically Defined Colon Cancer Antigen 8 (*SDCCAG8*, *P*_SZ_ = 1.68 × 10^−12^) was the most significantly associated of these genes.

TWAS was also performed for this subset of genes using both the brain and blood derived tissue panels. Of the genes tested, 26 and 18 models had sufficient overlapping SNP weights for the analysis considering brain and blood, respectively (Supplementary Table 7). Nine genes survived correction across both tissues. The most significant association with schizophrenia in the DLPFC construct for this subset of genes was increased predicted expression of *SDCCAG8*—*Z*_TWAS_ = 6.16, *P* = 7.21 × 10^−10^. Several of these genes remained significant after the application of both Bonferroni correction and conditional analysis for the DLPFC models—*CLNC3* (*Z*_TWAS_ = 5.36, *P* = 8.36 × 10^−8^), *SLC45A1* (*Z*_TWAS_ = −5.2, *P* = 1.97 × 10^−7^), *KCNN3* (*Z*_TWAS_ = −5.14, *P* = 2.63 × 10^−7^), *GIGYF1* (*Z*_TWAS_ = 4.59, *P* = 4.32 × 10^−6^) and *FAM114A2* (*Z*_TWAS_ = −4.51, *P* = 6.54 × 10^−6^). However, after conditional analysis considering proximally located associations, *SDCCAG8* was only marginally significant (*P* = 0.016), with another gene at this locus (*CEP170*) explaining the majority of the association. In addition, the two genes which survived correction for blood were also only marginally significant after a conditional test (*HVCN1*: *P*_Raw_ = 3.74 × 10^−7^, *P*_Conditonal_ = 0.011; *SBNO1*: *P*_Raw_ = 7.78 × 10^−7^, *P*_Conditonal_ = 0.025).

### Biologically constrained genes were enriched with common variation across multiple psychiatric disorders

**Fig. 3:**
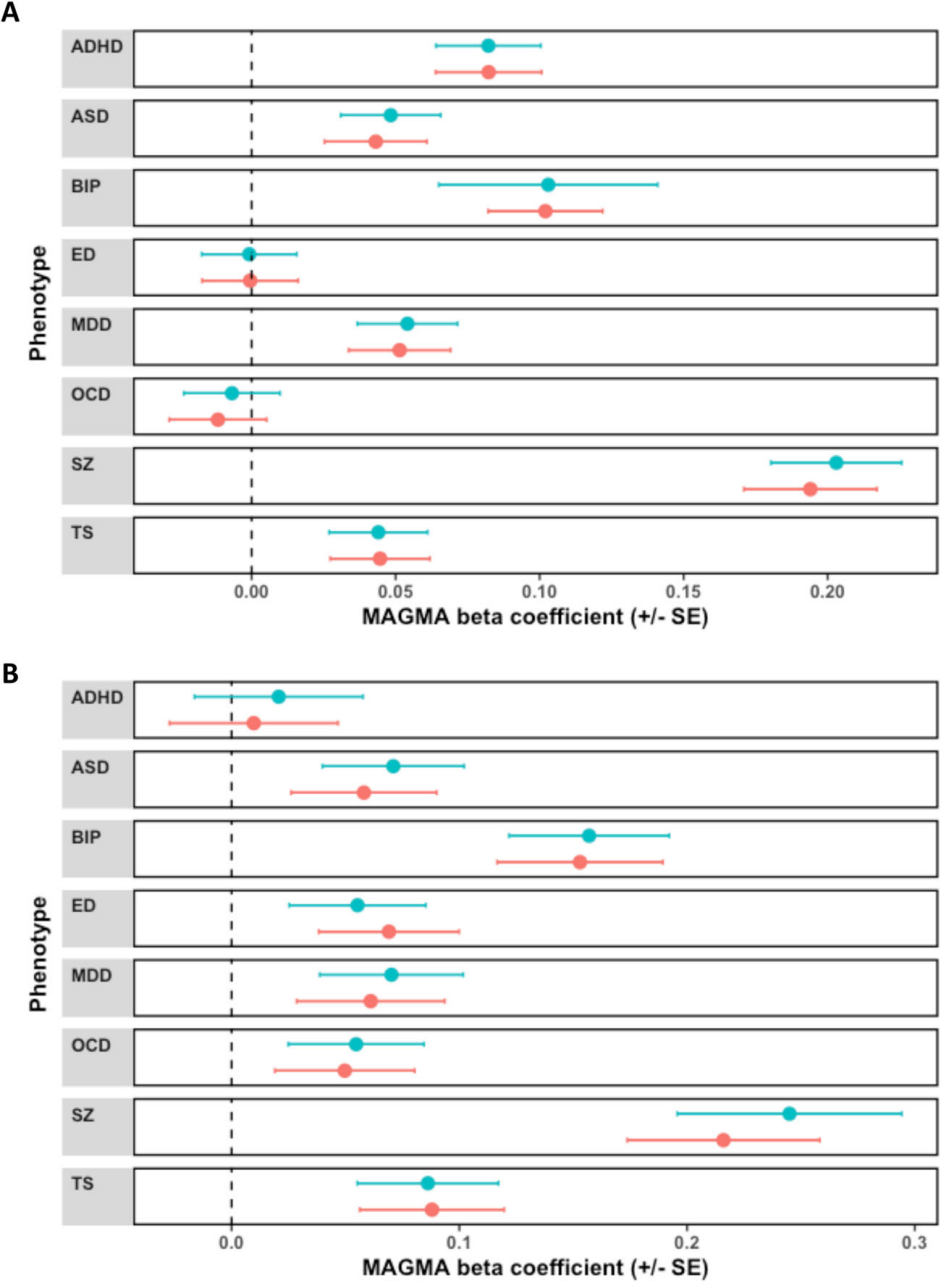
Enrichment of common variant associations within biologically constrained genes and FMRP target genes across different psychiatric disorders. Beta coefficient from MAGMA gene-set association model presented for each psychiatric phenotype, which tested association with **a** a set of genes which display intolerance to loss of function variation (probability of loss of function intolerant [pLI] ≥ 0.9, *N*_Genes_ = 3230), **b** targets of FMRP, *N*_Genes_ = 842. Error bars represent the upper and lower bounds of each coefficient relative to its standard error. Blue points indicative of the MAGMA model constructed without covariation for the expression of each gene in the cortex, while pink represents the models covaried for cortical gene expression.

We performed two gene-set association analyses, firstly, two hypothesis driven gene-sets associated with schizophrenia in the largest GWAS after multiple-testing correction were investigated in relation to the psychiatric disorders, and secondly, a data-driven approach using 7296 gene-sets from the MSigDB^23^. The hypothesis driven constructs were genes intolerant to loss of function variation (probability of loss of function intolerant [pLI] ≥ 0.9, *N*_Genes_ = 3230), and targets of the mRNA binding protein fragile X mental retardation protein (FMRP)^1,24,26^. Six of the eight disorders displayed some level of enrichment for common variant associations in mutation intolerant gene-set, with ED and OCD not significant (Fig. 3). Schizophrenia displayed the strongest association with biologically constrained genes (*β* = 0.203, SE = 0.0227, *P* = 1.94 × 10^−19^), followed by BIP (*β* = 0.103, SE = 0.0196, *P* = 7.24 × 10^−9^) and ADHD (*β* = 0.082, SE = 0.0182, *P* = 3.15 × 10^−6^). Genic constraint has previously been demonstrated to be related to gene expression—thus, as psychiatric risk genes are likely to have high neurological expression we repeated these analyses adjusted for gene-wise expression in the brain^1,24^. After covariation for the expression of each gene in the cortex, this signal remained at least nominally significant in all disorders except ED and OCD (Supplementary Table 8). BIP and schizophrenia demonstrated a notably strong signal for enrichment within the FMRP targets (Schizophrenia: *β* = 0.245, SE = 0.0352, *P* = 1.15 × 10^−9^, BIP: *β* = 0.157, SE = 0.0493, *P* = 3.93 × 10^−6^). The remaining phenotypes all demonstrated nominal association with the exception of ADHD, with only a minor effect of adjustment for gene-wise brain expression analogous to the models for the biologically constrained gene-set (Supplementary Table 9). Gene-set association analysis was then undertaken using 7296 hallmark, canonical, and gene ontology (GO) gene-sets from MSigDB. Fifteen of these gene-sets were associated with schizophrenia after Bonferroni correction (*P* < 6.8 × 10^−6^, Supplementary Table 10). When gene-set association was performed for the remaining seven phenotypes, the 15 schizophrenia-associated gene-sets did not survive Bonferroni correction for any disorder (Supplementary Table 11).

### Pairwise meta-analysis of schizophrenia with additional psychiatric disorders revealed novel gene-level associations

**Fig. 4:**
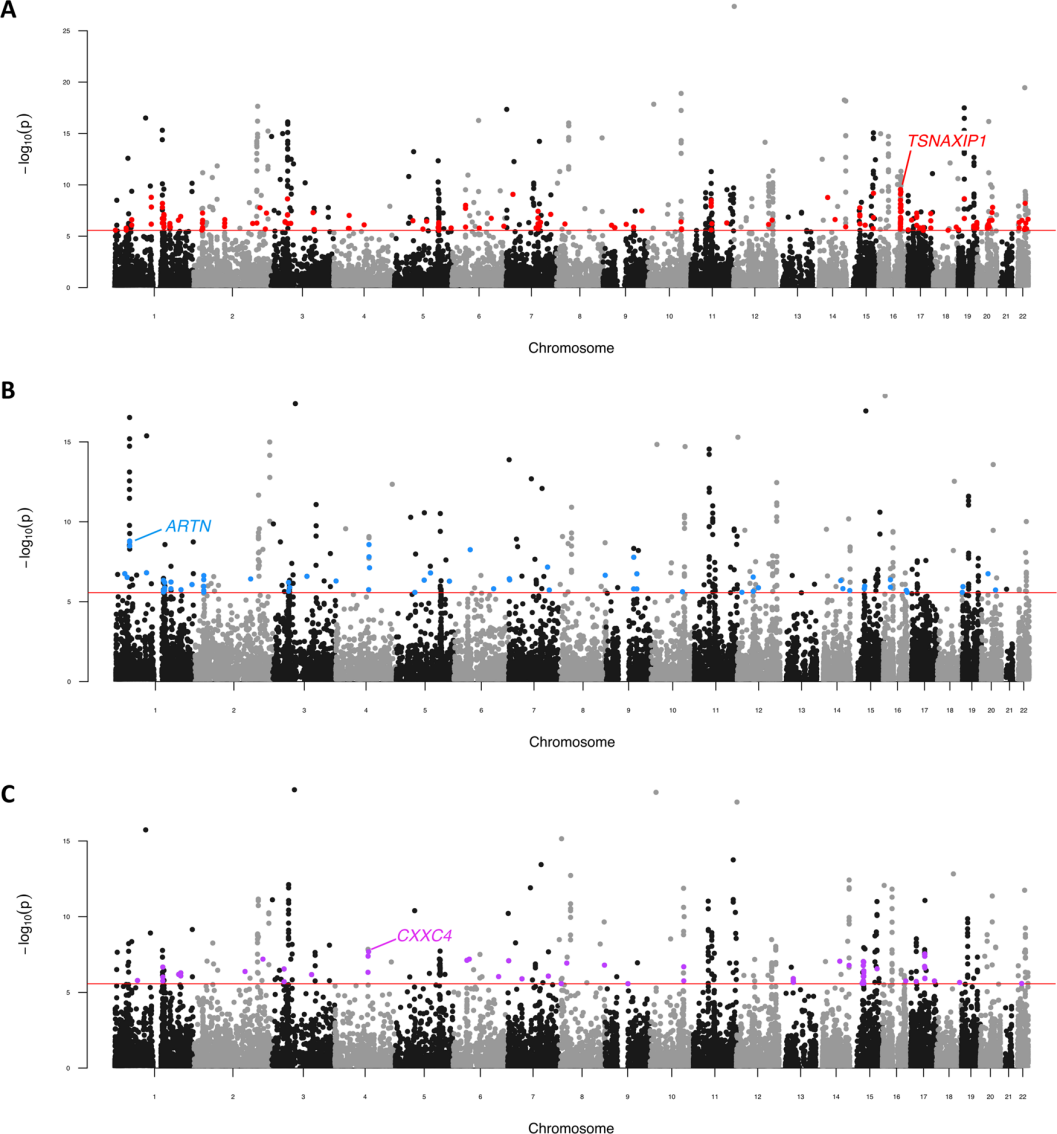
Pairwise genic meta-analysis of schizophrenia and other psychiatric disorders. Manhattan plot for each meta-analysis which displays the −log_10_-transformed *P* value for association for genes which were tagged by at least one SNP in the respective GWAS. The red line represents the Bonferroni threshold for multiple-testing correction (*P* < 2.7 × 10^−6^). Genes highlighted on each plot were not Bonferroni significant in the individual GWAS but obtained corrected significance in the meta-analysis, with the most significant of these genes labelled for each plot. **a** Schizophrenia (SZ) and bipolar disorder (BIP) genic meta-analysis, **b** Schizophrenia and attention-deficit/hyperactivity disorder (ADHD) genic meta-analysis. **c** Schizophrenia and autism-spectrum disorder (ASD) genic meta-analysis. Manhattan plots for the meta-analyses with MDD, TS, ED and OCD are presented in Supplementary Fig. 1.

Schizophrenia was meta-analysed at the gene-level in a pairwise fashion with the remaining psychiatric GWAS in order to identify genes which survived multiple-testing correction (*P*_Meta_ < 2.7 × 10^−6^) in each meta-analysis, but did not survive correction in the respective individual GWAS (2.7 × 10^−6^ < *P* < 0.05). All seven meta-analyses of schizophrenia and one other psychiatric disorder revealed at least one sub-threshold pleiotropic gene which satisfied the above criteria (Supplementary Table 12, Supplementary Fig. 1). The schizophrenia/BIP model yielded the largest number of novel genic associations (*N*_Novel_ = 175, lowest *P*: *TSNAXIP1*, *P* = 2.83 × 10^−10^—Fig. 4a). Thereafter, the schizophrenia/ADHD (*N*_Novel_ = 65, lowest *P*: *ARTN*, *P* = 1.6 × 10^−9^—Fig. 4b) and schizophrenia/ASD (*N*_Novel_ = 58, lowest *P*: *CXXC4*, *P* = 2.04 × 10^−8^—Fig. 4c) meta-analyses had the most novel genic associations. Several previously postulated psychiatric risk genes from candidate studies were revealed in these constructs, including neural cell adhesion molecule 1 (*NCAM1*) which was significant in the schizophrenia and BIP model^33,34^ and the Delta opioid receptor gene *OPRD1* which survived correction in the schizophrenia meta-analyses with ADHD and MDD, respectively^35,36^.

Across all seven meta-analyses there were 330 genes in total which only achieved corrected significance once schizophrenia was combined with another psychiatric trait. A notable proportion of this subset (*N* = 76) were identified across multiple meta-analyses, of which 20 survived correction in three pairwise models—for example, *CXXC4* which is a regulator of Wnt signalling^37^. Gene-set association analysis used the *Z*-scores derived from each meta-analysis for the data-driven gene-sets from MSigDB. All pairwise models demonstrated at least one gene-set which survived multiple-testing correction, largely recapitulating the gene-sets which were also significant in the univariate analysis of schizophrenia (Supplementary Table 13).

## Discussion

We investigated the common variant genomic architecture of schizophrenia relative to a range of psychiatric disorders. Previous work has suggested that there is a significant relationship between the genetics of schizophrenia and the spectrum of psychiatric phenotypes^38,39^, which is not unexpected given the analogous aspects of their respective clinical presentations. Moreover, a recent cross-disorder GWAS conducted by the PGC uncovered 109 variants with a significant effect on at least two psychiatric disorders^20^. We furthered this work by conducting gene-based analysis, which consolidated this aetiological overlap as schizophrenia shared several significant genic associations with other psychiatric disorders. It should be noted that this study employed a unified framework for defining genic boundaries across all phenotypes, and thus, the gene-based results may not exactly replicate the conditions implemented for the original GWAS studies which performed MAGMA, as a range of different gene definitions were used therein. Our boundary definition (5-kb upstream, 1.5-kb downstream) were conservative and may miss important regulatory regions, particularly as these regulatory elements can have distal effects as evidenced by chromosome conformation capture data^40^. We propose that further work is required to define the most appropriate boundaries, which could be adaptative for each gene and its respective regulatory architecture. Interestingly, the most strongly associated schizophrenia gene in this study, *CACNA1C*, was also strongly enriched with common variant associations for BIP. The Cav1.2 L-type voltage-gated calcium channel α1c subunit is encoded by *CACNA1C*, which is postulated to play an important role in synaptic plasticity^41–43^. It should be noted that a previous study implemented a TWAS-based approach using S-PrediXcan to uncover shared genes between schizophrenia, BIP, ADHD and MDD, with a variety of brain regions from GTEx, as well as SNP weights derived from blood, colon and adrenal gland GTEx tissue samples^44^. These data utilised a smaller sample size BIP GWAS than our study, however, *GNL3* was also associated with both schizophrenia and BIP in this study, albeit using a GTEx cerebellum SNP weight set. Further, we replicated the observed pleiotropic downregulation of *MED8* in schizophrenia and ADHD, while the previous study demonstrated this association with ADHD was also significant using adrenal gland tissue. Future analyses should also consider the shared genetic architecture between schizophrenia and other important related phenotypes, including substance use disorders, of which many display high rates of comorbidity and genetic correlation with schizophrenia^45–47^.

The majority of schizophrenia-associated genes were not significant for any other disorder after the application of multiple-testing correction. However, a large proportion of these genes displayed some degree of nominal significance (*P* < 0.05)—thus, we identified a subset of schizophrenia genes which displayed no evidence of association with any other psychiatric disorder (*P* > 0.05). This strict threshold was chosen as the sample sizes differ amongst the respective GWAS and future increases in sample sizes may yet reveal nominal *P* values which survive multiple-testing correction. The biological saliency of variation mapped to a subset of these genes was supported by TWAS. For instance, decreased predicted expression of the potassium channel *KCNN3* was associated with schizophrenia. Dopamine homoeostasis has been extensively linked to this protein, with blockade shown to increase spike firing of dopaminergic neurons, along with potentiated dopamine release^48,49^. Downregulation of *KCNN3*, therefore, is consistent with overarching dopamine hypotheses related to schizophrenia, supporting its relevance for pathogenesis of the disorder. An important caveat to these findings is that gene expression data utilised in this study for SNP weights was taken from samples which do not represent embryonic neurodevelopment or early post-natal timepoints. Given the importance of early development in neuropsychiatric disorders and the transient nature of gene expression across the lifespan, more efforts are needed to overcome the challenges involved in collecting post-mortem tissue from a variety of ages. Furthermore, the DLPFC was the only brain region analysed as it had the largest sample size and greatest number of *cis*-heritable genes available. This approach may have missed important biological insights from other brain regions. TWAS weights for different brain regions from GTEx have been used previously and future analyses could consider these disparate regions^44^, however, care should be taken given the differing sample sizes and number SNP weights available for these respective tissues.

The enrichment of common variant associations within genes under biological constraint was shown to be relatively ubiquitous across most of the psychiatric phenotypes considered in this study. These mutation intolerant regions of the genome are subject to purifying selection and, thus, it may appear contradictory that risk alleles for psychiatric disorders persist in these genes at common frequencies. It has been postulated that linkage between regions under repeated selection results in the loss of haplotypes, in turn, attenuating the strength of selection on individual sites^1,50,51^. This weakened selective pressure is theorised to account for the increase in frequency of these risk alleles through the action of enhanced genetic drift^50,52^. Therefore, this mechanism of common variant enrichment within constrained regions appears to be an important aspect of the genomic architecture of several psychiatric disorders which is shared with schizophrenia. These data also support a previous observation that highly constrained genes were more likely to be proximal to GWAS signals^24^, while Hussin et al. propose that regions with low recombination rates are enriched with constrained genes^53^. OCD did not display a significant association with this gene-set, as perhaps would be expected, however, the small sample size of its GWAS may account for this. Interestingly, more recent data have demonstrated that genes under biological constraint are depleted of eQTLs, and thus, disorder associated *cis*-regulatory variants in these genes may have greater impact^54^. Deleterious rare variation within these regions has also shown to be enriched for schizophrenia and ASD, consolidating the multi-faceted nature of genomic risk for these disorders^3,55^.

This study refines the nature of common variant informed genetic overlap between schizophrenia and other psychiatric conditions. A number of interesting pleiotropic candidates were revealed in this study which warrant further functional investigation to elucidate their significance to the respective phenotypes. For instance. *SORCS3*, the most pleiotropic schizophrenia-associated gene uncovered by these analyses due to its association with two of the other disorders considered, is implicated in a number of neurologically salient processes including modulation of synaptic depression and glutamate receptor functionality^56,57^. There are a number of important limitations to this study. Firstly, this work relies on the diagnostic definitions encompassed within each GWAS, however, the true incidence of psychiatric comorbidities amongst the respective study participants is unknown. Despite the uncertainty of psychiatric nosology, schizophrenia-associated genes involvement with another psychiatric GWAS does suggest that these shared genes may be particularly biologically salient. Further, the non-pleiotropic subset of genes identified in this study may be associated with other psychiatric phenotypes not considered in this study. We utilised common variant data for this study. While common variation is an integral component of psychiatric heritability, future cross-disorder investigation of genes and systems significantly enriched with rare loci will be integral to fully appreciate the spectrum of biology which constitutes the shared factors which exist between schizophrenia and these disorders. European GWAS data were chosen for these analyses as diverse well-powered summary statistics are still not readily available for many psychiatric traits. There are important ancestral differences between haplotype structure, and thus, it is essential that GWAS are more widely performed for non-European ancestral groups so that work of this fashion can be consolidated and leveraged in an inclusive manner. Finally, the principal multimarker test employed for gene discovery in this study, MAGMA, is based on *P*-value combination and assigns variants to genes using their genomic coordinates. This does not directly inform the effect size or functional significance of variation which constitutes these gene-based *P* values. TWAS helps to overcome this by assigning weights to SNPs based on the *cis*-heritability of their respective genes. However, TWAS relies on genotyped expression datasets of modest sample sizes, with many genes lacking a suitable imputed model of genetically regulated expression, and therefore, TWAS may be inherently conservative for gene identification. New methods for gene identification using *cis*-regulatory variants have been proposed such as eQTL-MAGMA (eMAGMA), whereby eQTLs are assigned to their target genes rather than using genomic position^58,59^, and future work could incorporate this method, along with other types of variants, into the gene-discovery step. Further study is required to advance these multimarker approaches such that statistical effect size and functional annotation beyond *cis*-regulatory annotations can be included. Enhanced models which integrate this information will facilitate the biological interpretation of schizophrenia-associated genes shared with other disorders.

## Supplementary information

Supplementary Figures

Supplemental Tables 1-8

Supplemental Tables 9-13

## Data Availability

Summary statistics are publicly available as described in the GWAS summary statistics section of the Materials and methods. All software is publicly available with versions and specific scripts also described above.

## Code availability

Command line inputs and code for figure generation in this study are available at https://github.com/Williamreay/Schizophrenia_cross_disorder_scripts.
