# Supplementary material for: Pairwise common variant meta-analyses of schizophrenia with other psychiatric disorders reveals shared and distinct gene and gene-set associations
Source: Transl Psychiatry. 2020 May 12;10:134. doi: 10.1038/s41398-020-0817-7 (PMC7217970; doi:10.1038/s41398-020-0817-7)
Supplement: Supplementary file 1 — Supplementary Figures [file 41398_2020_817_MOESM1_ESM.pdf]

Supplementary Figure 1

a

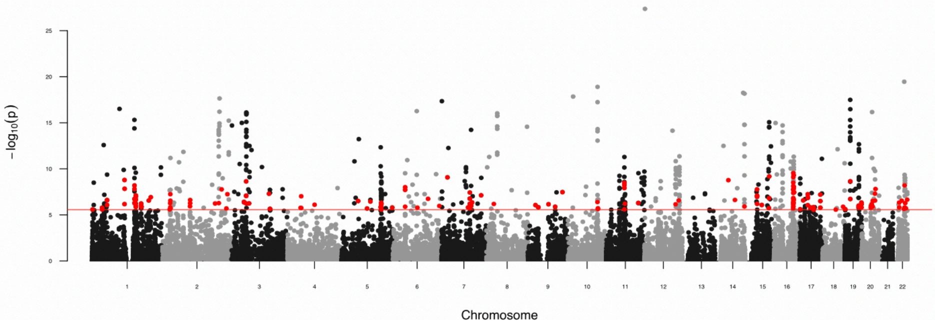

b

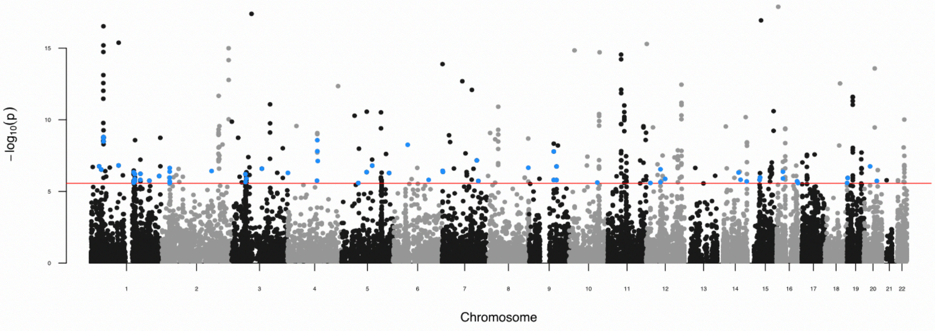

c

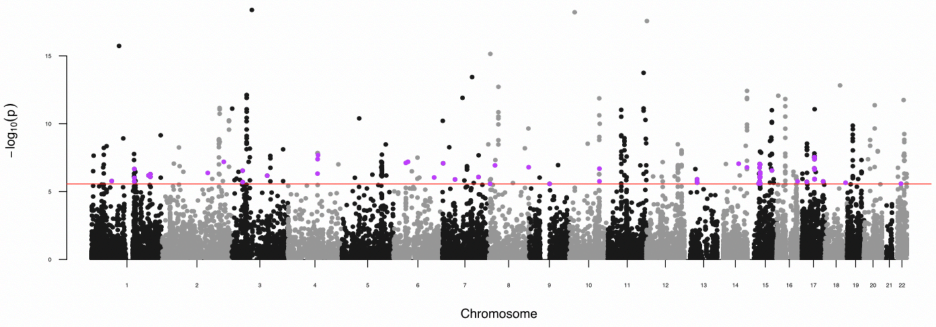

d

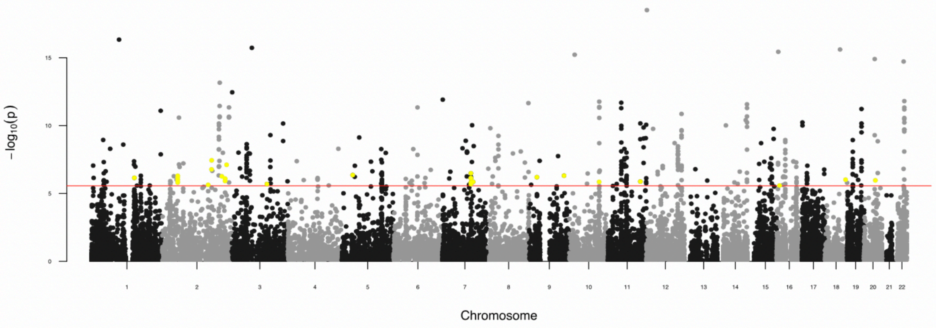

$-\log_{10}(p)$
0
5
10
15
1 2 3 4 5 6 7 8 9 10 11 12 13 14 15 16 17 18 19 20 21 22
Chromosome

e

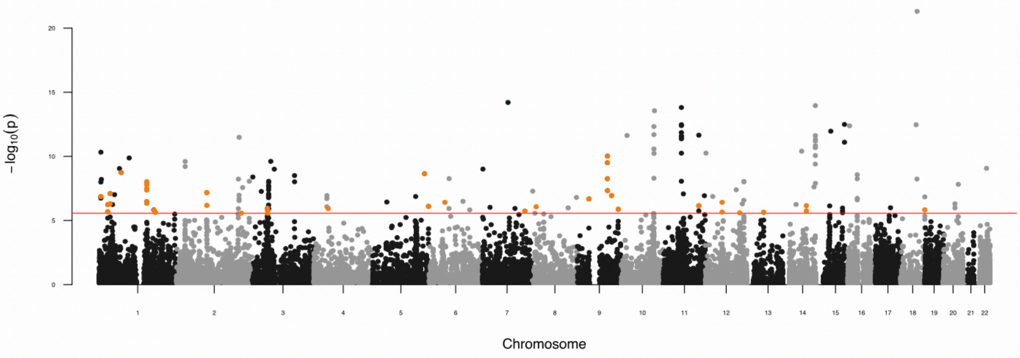

$-\log_{10}(p)$
0
5
10
15
20
1 2 3 4 5 6 7 8 9 10 11 12 13 14 15 16 17 18 19 20 21 22
Chromosome

f

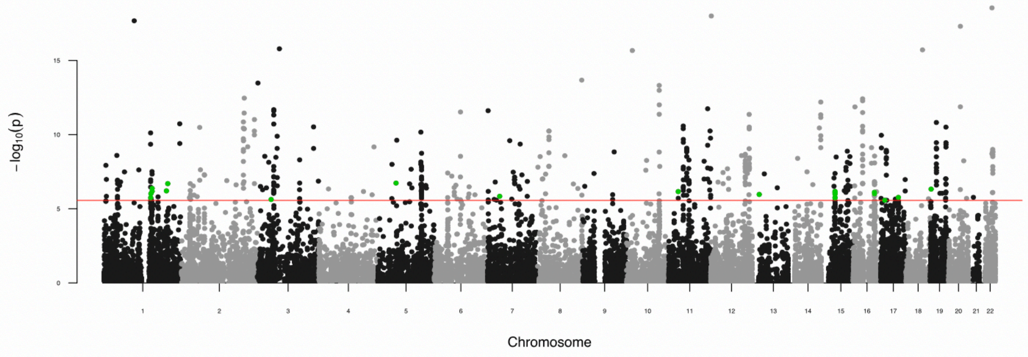

$-\log_{10}(p)$
0
5
10
15
1 2 3 4 5 6 7 8 9 10 11 12 13 14 15 16 17 18 19 20 21 22
Chromosome

g

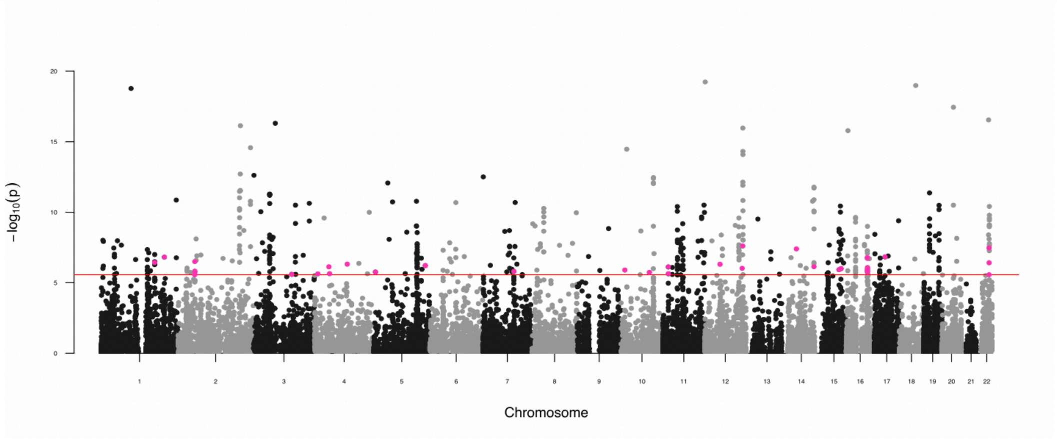

Supplementary Figure 1. Pairwise genic meta-analysis of schizophrenia and other psychiatric disorders. Manhattan plot for each meta-analysis which displays the $-\log_{10}$ transformed *P* value for association for genes which were tagged by at least one SNP in the respective GWAS. The red line represents the Bonferroni threshold for multiple testing correction ($P < 2.7 \times 10^{-6}$). Genes highlighted on each plot were not Bonferroni significant in the individual GWAS but obtained corrected significance in the meta-analysis. (**a**) Schizophrenia (SZ) and Bipolar Disorder (BIP) genic meta-analysis, (**b**) Schizophrenia and Attention Deficit/Hyperactivity Disorder (ADHD) genic meta-analysis. (**c**) Schizophrenia and Autism Spectrum Disorder (ASD) genic meta-analysis, (**d**) Schizophrenia and Eating Disorder (ED) genic meta-analysis, (**e**) Schizophrenia and Major Depressive Disorder (MDD) genic meta-analysis, (**f**) Schizophrenia and Obsessive-Compulsive Disorder (OCD) genic meta-analysis, (**g**) Schizophrenia and Tourette's Syndrome (TS) meta-analysis.

BIP

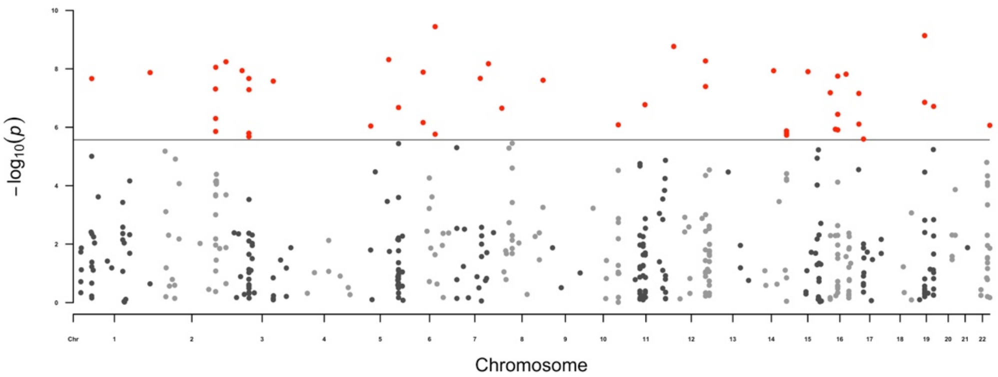

MDD

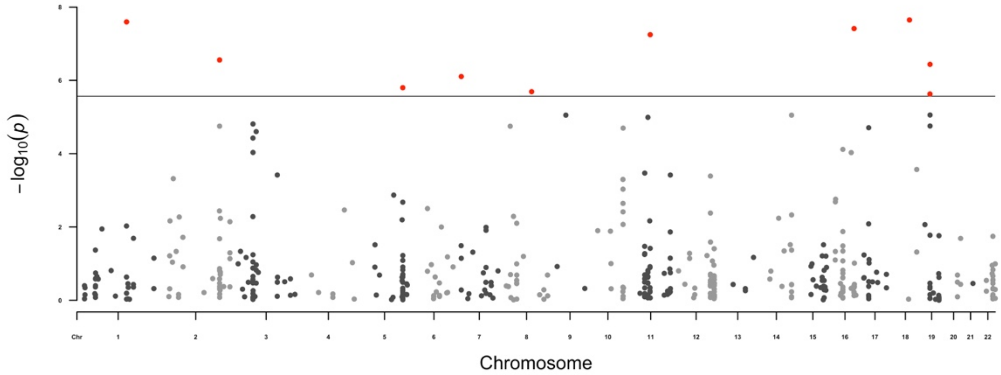

ADHD

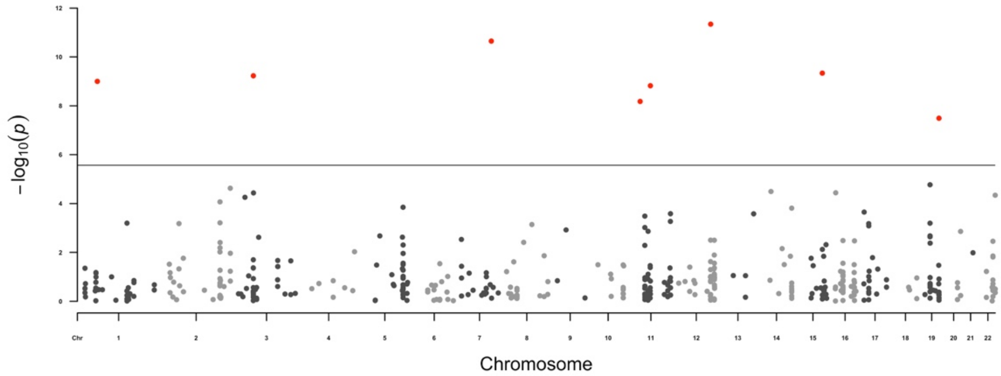

ASD

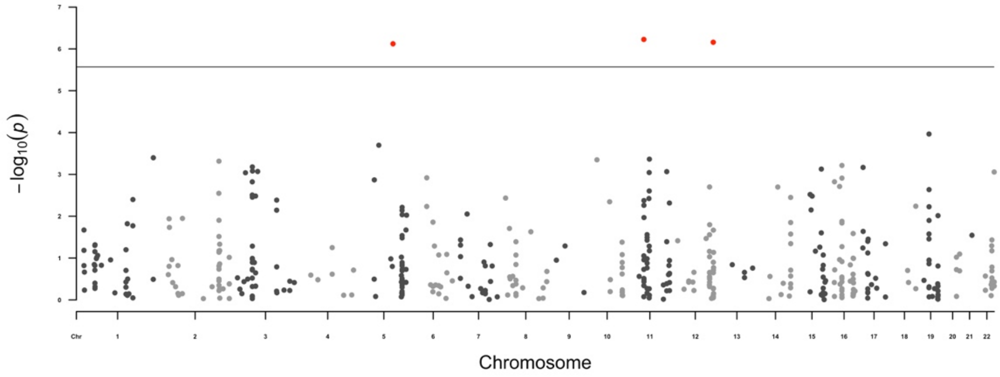

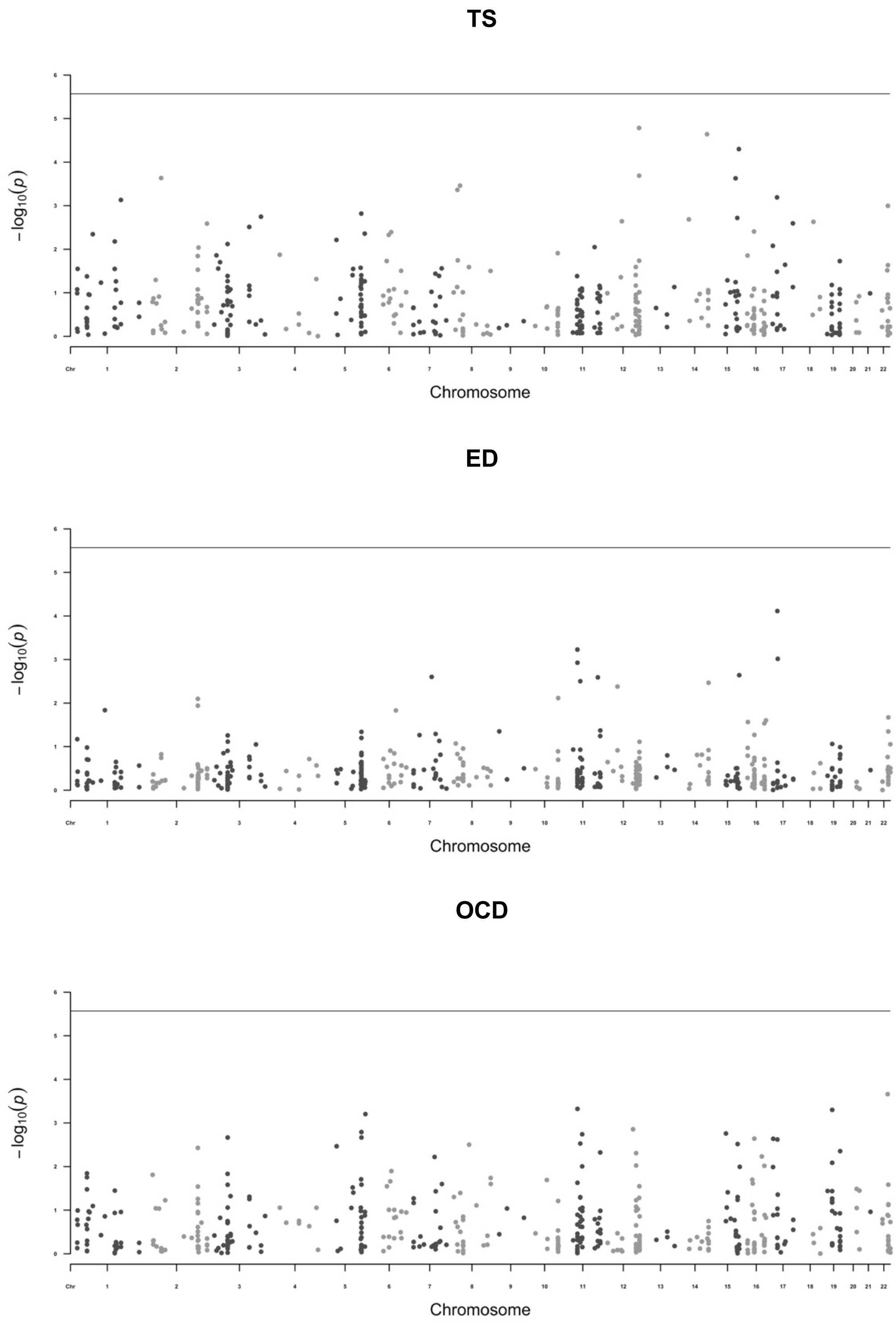

Supplementary Figure 2. Schizophrenia associated genes shared with other psychiatric disorders. Association of Bonferroni significant schizophrenia genes with seven other psychiatric disorders. Results presented as Manhattan plot of the -$\log_{10}$ *P*-value of association for each gene per disorder. Significant schizophrenia genes by MAGMA which survive correction in each disorder are highlighted red ($P < 2.7 \times 10^{-6}$).
